# Differential diagnosis between kaposi sarcoma-associated herpesvirus cytokine syndrome and hemophagocytic lymphohistiocytosis

**DOI:** 10.1007/s15010-024-02252-7

**Published:** 2024-03-29

**Authors:** Mario Luppi, Andrea Cona, Patrizia Barozzi, Giovanni Riva, Alessandra Mularoni

**Affiliations:** 1 Section of Hematology, Department of Medical and Surgical Sciences, University of Modena and Reggio Emilia, AOU Modena, Modena, Italy; 2 Unit of Infectious Diseases, ISMETT-IRCCS Istituto Mediterraneo per i Trapianti e Terapie ad Alta Specializzazione, Palermo, Italy; 3 Diagnostic Hematology and Clinical Genomics Laboratory, Department of Laboratory Medicine and Pathology, AUSL/AOU Modena, Modena, Italy

To the Editor,

we read with interest the paper by Liapis et al., [1] claiming to report the occurrence of a variant of Kaposi’s sarcoma-associated herpesvirus (KSHV) inflammatory cytokine syndrome (KICS) in two human herpesvirus-8 seropositive (HHV-8) elderly Greek men, without recognizable causes of immunosuppression. We tend to dispute such a diagnosis for several reasons.

First, a diagnosis of KICS has been defined in patients who showed elevated KSHV/HHV-8 viral loads, elevated C-reactive protein (CRP), no evidence of Multicentric Castleman Disease (MCD), and at least two serious clinical abnormalities in three categories (symptoms, abnormal laboratory findings, and abnormal radiographic findings) [2] but signs of hemophagocytosis have not been included among the criteria to be fulfilled for the case definition of KICS. Prominent proliferation of hemophagocytic histiocytes ingesting hematopoietic cells have clearly been shown in both patients’ bone marrow [1]. Although hemophagocytosis in tissue biopsy is not exclusively seen in Hemophagocytic Lymphohistiocytosis (HLH), hemophagocytosis in bone marrow or spleen or lymph nodes is among the well-recognized diagnostic criteria to be fulfilled to establish a diagnosis of HLH [3].

Second, the authors tend to exclude a diagnosis of KSHV/HHV-8 associated MCD, because of the absence of lymph node enlargement in patient 1 and no lymphadenopathy, and unremarkable positron-emission tomography-computed tomography in patient 2. However, in the absence of histopathologic examination of the enlarged spleen, a diagnosis of MCD cannot be formally ruled out in patient 1.

Third, most importantly, the clinical presentation and laboratory abnormalities are clearly diagnostic of HLH [3]. Relevant to this, the probability of HLH using the H score was 98% in both reported patients [4]. We already reported the concomitant/subsequent occurrence of KICS and HLH, following primary HHV-8 infection in a kidney-liver recipient, successfully treated with a combination of rituximab, antivirals, and modification of the immunosuppressive regimen [5]. Rather surprisingly, the authors consider a diagnosis of HLH “presumed” because treatment with glucocorticoids and intravenous immunoglobulins were ineffective [1]. However, it should be noted that treatment in adults with HLH cannot be standardized and need to be based on underlying conditions and HLH-initiating triggers (infection, malignancy, autoimmune/autoinflammatory, etc.) [3]. Of note, the treatment algorithm for adult patients with herpesvirus associated HLH may include not only immunoglobulins but also antivirals, and, in severe cases, following the modified HLH 94 protocol, also dexamethasone and etoposide, and/or rituximab, when Epstein-Barr virus and KSHV/HHV-8 are the recognized infectious triggers [3, 5].

Despite the use of rituximab combined with cyclophosphamide, adriamycin, vincristine, and prednisone was successful in both patients, without apparent toxicity, at least in the short-term, in principle, this therapeutic option is questionable in these patients. It should be noted that this therapeutic regimen is commonly used in patients with B cell non-Hodgkin lymphomas, but neither recommended nor, so far, being used for reported cases of either KICS or EBV/KSHV/HHV-8 induced HLH [3, 5]. Moreover, currently, the standard treatment for HHV-8 positive MCD patients positive for human immunodeficiency virus is rituximab in low-risk disease, with the addition of either etoposide, in patients with a poor performance status (ECOG < 2) or end-organ damage (involvement of organs other than spleen and lymph nodes, hemophagocytic syndrome, or hemolytic anemia) or pegylated liposomal doxorubicin, in the presence of concomitant Kaposi Sarcoma. Thus, further controlled studies on larger series of patients are warranted before the data reported by the authors may be generalizable and the use of such a chemotherapeutic regimen, combined with rituximab, is consistently proposed in the treatment of non-neoplastic KSHV/HHV-8 related clinical manifestations.

In conclusion, we recommend that more caution is needed to define a specific diagnosis of KSHV/HHV-8 related non-neoplastic related diseases. Efforts should be made to promptly recognize KSHV/HHV-8 induced HLH to allow more tailored, effective but potentially less toxic treatments. Studies, assessing interleukin-6/interleukin-10 and other cytokine signatures, are also needed to better characterize the spectrum and distinct features of KSHV/HHV-8-related non-neoplastic diseases also in HIV-negative individuals, before proposing to revise KICS criteria or “simply” recognizing new clinical variants of KICS. The diagnostic criteria for KICS and HLH lack specificity and the demarcation between KICS and HLH may be blurred. More research is needed to differentiate between HLH and KICS.
